# Sanger validation of WGS variants

**DOI:** 10.1038/s41598-025-87814-x

**Published:** 2025-01-29

**Authors:** Arina Kopernik, Mariia Sayganova, Gaukhar Zobkova, Natalia Doroschuk, Anna Smirnova, Daria Molodtsova-Zolotukhina, Olesya Sagaydak, Oxana Ryzhkova, Sergey Kutsev, Olga Groznova, Lyusya Melikyan, Elizaveta Bondarchuk, Mary Woroncow, Eugene Albert, Viktor Bogdanov, Pavel Volchkov

**Affiliations:** 1 Federal Research Center for Innovator and Emerging Biomedical and Pharmaceutical Technologies, Moscow, Russia 125315; 2 Evogen LLC, Moscow, Russia; 3 Research Centre for Medical Genetics, Moscow, Russia 115478; 4 Lomonosov Moscow State University, Moscow, Russia; 5 Veltischev Research and Clinical Institute for Pediatrics and Pediatric Surgery on the Pirogov Russian National Research Medical University of the Ministry of Health of the Russian Federation, Moscow, Russia; 6 Charity Fund for Medical and Social Genetic Aid Projects «Life Genome», Moscow, Russia; 7 The Pirogov Russian National Research Medical University of the Ministry of Health of the Russian Federation, Moscow, Russia

**Keywords:** Genetic variation, Sequencing, Next-generation sequencing

## Abstract

With the development of next-generation sequencing (NGS) technologies it became possible to simultaneously analyze millions of variants. Despite the quality improvement, it is generally still required to confirm the variants before reporting. However, in recent years the dominant idea is that one could define the quality thresholds for “high quality” variants which do not require orthogonal validation. Despite that, no works to date report the concordance between variants from whole genome sequencing and their gold-standard Sanger validation. In this study we analyzed the concordance for 1756 WGS variants in order to establish the appropriate thresholds for high-quality variants filtering. Resulting thresholds allowed us to drastically reduce the number of variants which require validation, to 4.8% and 1.2% of the initial set for caller-agnostic (DP, AF) and caller-dependent (QUAL) thresholds, respectively.

**Supplementary Information:**

The online version contains supplementary material available at 10.1038/s41598-025-87814-x.

## Introduction

Next Generation Sequencing (NGS) is a powerful method of genome analysis. Since the introduction of the technique, according to the American College of Medical Genetics (ACMG) guidelines, discovered variants were required to be validated with an orthogonal method before reporting^1^. Usually, it was done by Sanger sequencing. As NGS technologies have matured, and the volumes of sequenced samples increased, the question of the need for such validation arose. Several studies have reported small panels and exome data results with up to thousands of variants analyzed^2–5^, which in general showed high (91.29%–98.7%) concordance between Sanger and NGS, reaching 100% for “high quality” (HQ) variants. Hence, in recent publications^6,7^ the recommendations were somewhat relaxed: each laboratory should either establish a confirmatory testing policy for the variants or continue working with orthogonal confirmation.

Based on the abovementioned studies, the thresholds for the variant parameters were suggested in different publications as minimum 100–500 quality score (QUAL) and 20-100 coverage depth (DP)^8–10^ (Table 1). The most recent study^11^ concerning short variants reported that high quality variants may be considered with FILTER = PASS, QUAL ≥ 100, DP ≥ 20 and allele frequency (AF) ≥ 0.2. However, these recommendations, derived from the panels and exomes, seem to be of limited use for the whole genome sequencing, as its mean depth of coverage is usually around 30x.

**Table 1:** Summary of previous studies.

Study	NGS type (variants with Sanger validation)	Technology	Mean sequencing depth	Recommendations
Sikkema-Raddatz et al.^3^	Targeted panel (168 variants)	SureSelect, MiSeq (Illumina)	257.5	DP ≥ 30, AF ≥ 0.2
Nelson et al.^5^	Targeted panel (296 variants)	Tru Sight One, SureSelect, HiSeq (Illumina)	Not stated	Filter = PASS, DP ≥ 20, QUAL > 300, AF for heterozygous = 0.3–0.6, AF for homo/hemizygous > 0.9
Yohe et al.^12^	Targeted panel (30 variants)	SureSelect, HiSeq 2000 (Illumina)	Not stated Minimum ≥ 20x	DP ≥ 20
Baudhuin et al.^8^	Targeted panel (1204 variants)	SureSelect, GAIIx or MiSeq	> 100x	DP ≥ 100, QUAL ≥ 20, Flanking regions mean base quality score > 15
Beck et al.^9^	Clinical Exome (5800 variants)	SureSelect or TruSeq, GAIIx or HiSeq 2000 sequencer (Illumina)	Not stated	MPG score > 10
Mu et al.^4^	Targeted panel (7845 variants)	HiSeq2500 or NextSeq500	441x	DP ≥ 100 AF ≥ 0.4
Zheng et al.^2^	Targeted panel, Whole Exome (7601 variants)	HiSeq2500, BGISEQ-500	Differs for each panel and sequencer	DP ≥ 35, AF ≥ 0.35
De Cario et al.^13^	Targeted panel (945 variants)	SureSelect, HaloPlex, MiSeq (Illumina)	173x for SureSelect, 1100x for HaloPlex	QUAL ≥ 30 DP ≥ 30*
Arteche-López et al.^11^	Clinical Exome (1109 variants)	TruSight (Illumina), NextSeq (Illumina)	ranged from 20x to 361x, with a mean of 107.74x	FILTER = PASS, QUAL ≥ 100 DP ≥ 20 AF ≥ 0.2
Current study	Whole Genome (1756 variants)	DNBSEQ-G400 DNBSEQ-T7	34.1x	Caller-agnostic: DP ≥ 15, AF ≥ 0.25, Haplotype Caller v4.2: QUAL ≥ 100

*Paper is not designed to identify quality thresholds. Hard filtering with these parameters was applied yielding 100% variants with these parameters being confirmed.

During our research, we could not encounter studies reporting validation results on the whole genome data, and the minority of all the reported data considers BGI technological platform. The aim of this study was to perform the analysis of the quality control parameters for 1756 potentially causative variants derived from WGS data of 1150 patients. Each of these variants was validated by Sanger sequencing. Based on this analysis we suggest a set of quality filters capable of separating variants which require Sanger validation from the “high quality” variants (the ones which do not require one). Implementing these metrics into routine practice will greatly reduce the need for Sanger sequencing—in our case, to 1.2% of the initial set—therefore decreasing time and cost of overall clinical WGS analysis.

## Results

### Dataset

A cohort of 1150 WGS samples was analyzed to find the cause of the disease and to identify carriers of potentially pathogenic variants. In total, 1756 variants were subjected to further validation, out of them 1555 SNVs (181 in introns, 1374 in exons) and 201 INDELS (20 in introns and 181 in exons). Mean coverage of the samples was 34.1x, ranging from 20.57x to 48.64x. Mean coverage depth (DP) of the variants was 33 (3–81), mean quality (QUAL) was 492 (30–2106).

All 1756 selected variants were subjected to validation by Sanger sequencing. In 5 (0.28%) cases WGS data did not match Sanger data, demonstrating 99.72% concordance.

### Optimal thresholds selection

Quality parameters were compared with the thresholds in the published data to demonstrate whether they are applicable to WGS data (Table 2). Out of these, one of the least stringent threshold sets in terms of the coverage depth belongs to the most recent manuscript^11^. These thresholds (FILTER = PASS, QUAL ≥ 100, DP ≥ 20, AF ≥ 0.2) allowed to filter out 210 “low-quality” variants, 5 of which were unconfirmed, resulting in 2.4% precision and outperforming other published thresholds.

**Table 2:** Classification statistics based on different thresholds.

Quality thresholds	“High quality” variants (unconfirmed variants)	“Low quality” variants (unconfirmed variants)	Test #1 “LQ bin identifies unconfirmed variants”		
			Sensitivity (Recall)	Precision	F_1_-score
DP ≥ 35, AF ≥ 0.35^2^	738 (0)	1018 (5)	100%	0.5%	0.010
DP ≥ 30 QUAL ≥ 30^13^	1088 (0)	668 (5)	100%	0.7%	0.015
DP ≥ 20^12^	1549 (1)	207 (4)	80.0%	1.9%	0.038
DP ≥ 20 AF ≥ 0.3 FILTER = PASS QUAL > 300^5^	1357 (0)	399 (5)	100%	1.3%	0.025
DP ≥ 20 AF ≥ 0.2 FILTER = PASS QUAL ≥ 100^11^	1546 (0)	210 (5)	100%	2.4%	0.047
DP ≥ 20 AF ≥ 0.2	1547 (0)	209 (5)	100%	2.4%	0.047
DP ≥ 15 AF ≥ 0.25	1672 (0)	84 (5)	100%	6.0%	0.112
QUAL ≥ 100	1735 (0)	21 (5)	100%	23.8%	0.385

**Fig. 1:**
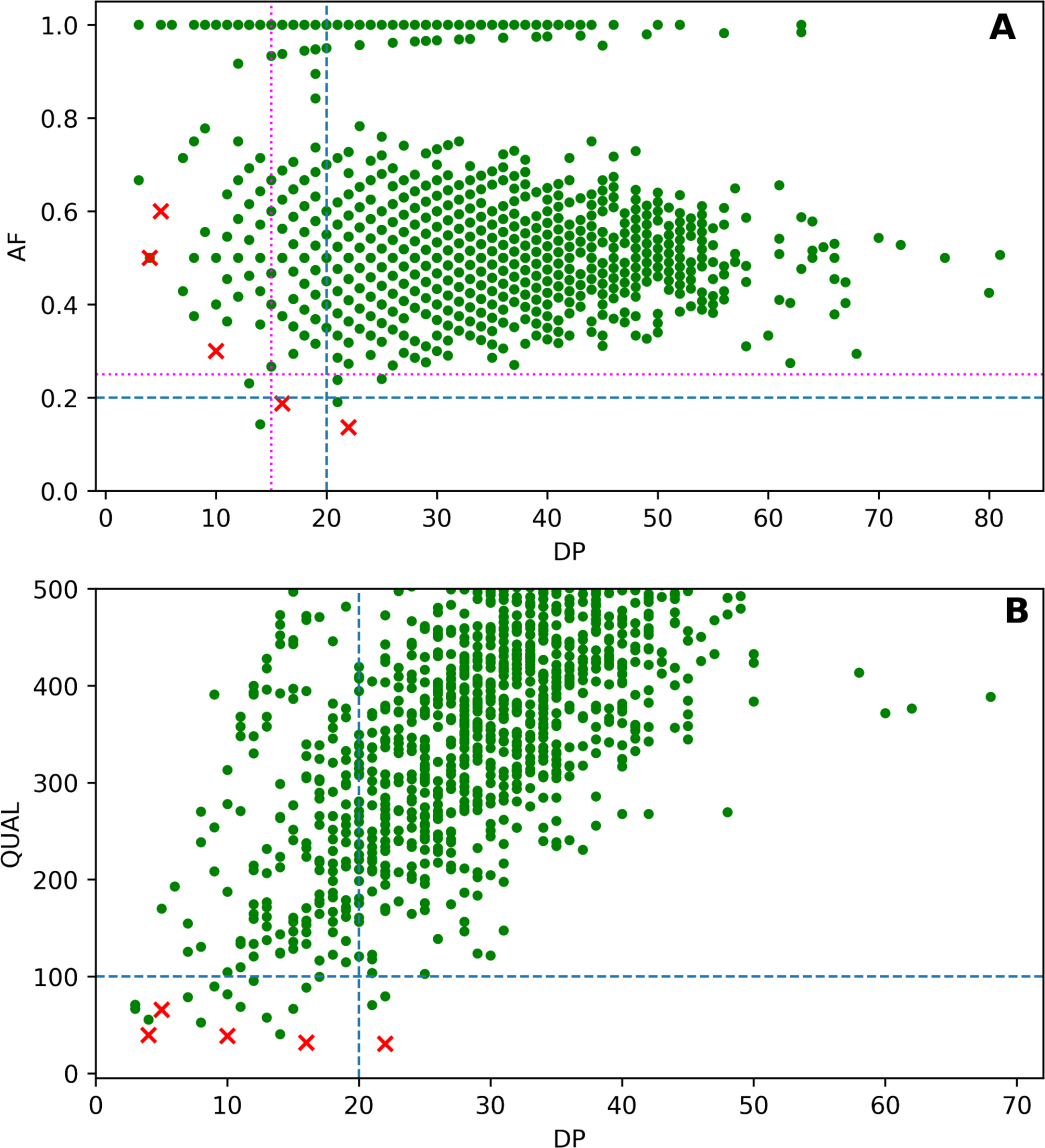
(**A**) Distribution of confirmed and unconfirmed variants depending on AF and DP parameters. (**B**) Distribution of confirmed and unconfirmed variants depending on QUAL and DP parameters. Green points represent confirmed variants and red X-s represent unconfirmed variants. Blue lines represent thresholds from recent WES study^11^, magenta line represents caller-agnostic quality thresholds suggested in this work (DP ≥ 15, AF ≥ 0.25).

It is possible to divide presented thresholds into two groups: caller-agnostic (AF and DP) and caller-specific (QUAL) parameters based on whether they depend on variant calling instrument or not. Speaking of caller-agnostic thresholds, variants with allele frequency (AF) parameter ≥ 0.2 and DP ≥ 20 alone demonstrated 100% concordance with Sanger data and similar precision and F_1_-score. It was possible for our dataset to lower DP parameter to 15 and increase AF to 0.25 in order to increase precision from 2.4% (210 low-quality variants, 5 of them unconfirmed) to 6.0% (84 low-quality variants, 5 of them unconfirmed) without losing sensitivity (Fig. 1A).

As for quality parameter (QUAL), it can be analyzed separately as a caller-specific threshold. All the variants with QUAL of 100 and above demonstrated 100% concordance with Sanger data, while 5 out of 21 variants with QUAL lower than 100 were unconfirmed which resulted in 23.8% precision (Fig. 1B).

### Application of the thresholds to the previous datasets

Out of the datasets presented in Table 1, the one from Zheng et al.^2^ is the only one which is simultaneously readily available, contains of the comparable number of variants, and has DP and AF data for both confirmed and unconfirmed ones. Caller-agnostic thresholds presented in Table 2 were therefore applied to this dataset with the results presented in Table S3 in the Supplement.

Firstly, one must note the fragility of the quality thresholds (DP ≥ 35, AF ≥ 0.35) presented in the original paper^2^. While these thresholds yield 100% sensitivity, lowering the AF parameter to ≥ 0.34 already results in 4 unconfirmed variants in the “high quality” (HQ) bin with overall sensitivity of 98.3%. Caller-agnostic parameters, suggested in this work (DP ≥ 15, AF ≥ 0.25), outperform the original thresholds in terms of F_1_-score (0.760 vs. 0.526) due to the increased precision (67.3% vs. 35.6%), albeit at the cost of sensitivity (87.3%, with 30 unconfirmed variants in the HQ bin).

Efficacy of the caller-agnostic thresholds from this work in application to the dataset depends significantly on the exact enrichment panel used in the original study (Table S4, Figures S2-S3), with the best results achieved for the hereditary deafness (HD) panel (96.7% sensitivity, 90.7% precision, F_1_-score 0.937, 1632 total variants with 2 unconfirmed variants in the HQ bin). Notably, sensitivity of the thresholds tends to decrease with the increase in panel size, with the worst result achieved for the exome panel BGI_Exo (75.0% sensitivity, 70.6% precision, F_1_-score 0.727, 855 total variants with 16 unconfirmed variants in the HQ bin).

## Discussion

The main aim of the validation policy for NGS data is to minimize the amount of additional testing without lowering the quality of a final result. Hence, the goal of the study was to identify a set of thresholds which robustly separate high quality WGS variants from the ones that require validation with appropriate sensitivity and precision.

According to Table 2, previously suggested threshold values^2,5,11,13^ work reasonably well on WGS data, filtering out all the unconfirmed variants into the “low quality” (LQ) bin. However, because of the differences in average sequencing depth between WES and WGS, the threshold of DP ≥ 20 is too high for WGS data, hence the precision of such approach is lower than one might expect (up to 2.4%). Additionally, QUAL and FILTER parameters are caller-specific and therefore the values are not strictly comparable between bioinformatic pipelines.

Based on our data, better results can be obtained using only caller-agnostic parameters of DP and AF. DP ≥ 15 and AF ≥ 0.25 achieve similarly sensitive results, filtering out all 5 unconfirmed variants into the LQ bin while shrinking the bin itself 2.5 times, which, if implemented as an actual policy, would reduce the confirmatory testing cost accordingly.

These caller-agnostic thresholds work reasonably well on the existing panel/exome datasets as well, achieving up to 97% sensitivity depending on the exact enrichment panel used. However, false positive variants end up in the HQ bin defined with the AF ≥ 0.25 threshold regardless of the coverage depth, which ranges from to 16 to 250, and the number of these variants increases with the increase in the panel size (Table S4, Figure S3). This trend might be explained by the PCR and/or enrichment biases which leads to the unequal representation of alleles. The absence of such variants in our dataset is also consistent with this explanation since the dataset is acquired using PCR-free WGS protocol.

An even greater result in terms of precision for the WGS data can be achieved using the QUAL parameter alone. Setting a single QUAL > 100 threshold achieves 23.8% precision without the reduction in sensitivity for the unconfirmed variants, which shrinks the LQ bin size to less than 2% of the total dataset. This result is understandable since the QUAL parameter itself encompasses a complex set of rules aimed at providing an evaluation of confidence in the presence of the variant at a given site. Importantly, we would not recommend a direct transfer of this threshold to different callers apart from the one used in this work (HaplotypeCaller v.4.2).

One must note that even for our best performing classification the LQ bin contains more than 75% of the validated variants. Therefore, such variants must not be hard filtered without validation in one way or another.

Apart from Sanger validation a consensus approach using another variant caller can be suggested. We have evaluated this approach by calling variants with QUAL < 100 with DeepVariant v1.5. Two out of five (40%) unconfirmed variants were still called by DeepVariant while 5/16 (31%) of the confirmed ones were missed by the caller, giving F_1_-score of 0.76 for such validation and indicating the need for the careful practical evaluation of such techniques which is beyond the scope of the current work.

## Conclusions

Our study demonstrates that previously suggested thresholds (DP ≥ 20, AF ≥ 0.2, QUAL ≥ 100) work reasonably well for WGS data separating the false positive variants with 100% sensitivity and 2.4% precision. However, for WGS we suggest lowering the DP requirements for “high quality” variants, which achieve, in our case, 6.0% precision with the same sensitivity for caller-agnostic thresholds (DP ≥ 15, AF ≥ 0.25). Additionally, QUAL is shown to be an independent classification parameter, which alone, for our dataset, can separate false positive variants with much greater precision of 23.8% using QUAL ≥ 100 threshold for HaplotypeCaller V4.2 thus tremendously reducing the need for validation.

## Materials and methods

### Sample acquisition

Venous blood samples for whole genome sequencing were collected from patients (N = 1150) during their testing at Evogen LLC. All participants gave an informed consent to Evogen LLC. The study was reviewed and approved by the Research Ethics Committee of Evogen LLC and fulfilled the principles of the Declaration of Helsinki.

### Samples and library preparation

Library preparation was performed using PCR-free enzymatic shearing protocol (MGIEasy FS PCR-Free DNA Library Prep Kit, MGI, China). Whole genome sequencing of 1150 patients in PE150 mode was performed using DNBseq-T7 and DNBseq-G400 (MGI, China) sequencers at EVOGEN LLC laboratory (Moscow, Russia). All the experimental stages were conducted in accordance with the manufacturer’s standard protocols. The average whole genome sequencing depth was 30x.

### Bioinformatics pipeline

The variant calling of the genetic variants was conducted using bioinformatics analysis accelerators (EVA Pro (EVOGEN, Russia). Raw reads were trimmed with fastp (0.23.1), mapped to the reference human genome (hg38) with minimap2 (2.17). Calling was performed after base recalibration with Haplotype Caller (4.2) in a single sample mode according to GATK best practice guidelines or with DeepVariant v1.5 (for low quality variants).

### Variant selection

After the bioinformatic analysis the data were reviewed by clinical variant scientists and geneticists in the search for the disease-causing variants according to the ACMG Standards and Guidelines, considered variants were pathogenic, likely pathogenic and uncertain significance. Candidate variants for the reporting were assigned to the lab for Sanger sequencing validation.

### Sanger sequencing

Causative genetics variants revealed by WGS were validated by Sanger sequencing at EVOGEN LLC. Sample DNA extraction was performed using QIAamp DNA blood Mini Kit (QIAGEN, Germany) / MGIEasy Magnetic Beads (MGI, China) by standard manufacturer’s protocols. Purified PCR products were sequenced using the BigDye Terminator Kit v3.1 and ABI 3500 Genetic Analyzer (Applied Biosystems, United States) by standard manufacturer’s protocols. The analysis of the sequenced data was performed using Variant Reporter Software v3.0 (Applied Biosystems, United States).

### Data analysis

Information on quality, allele frequency and coverage depth was extracted from vcf files. The dataset was analyzed with the custom python scripts.

For the evaluation of the thresholds the test (Test #1) was formulated as “Low quality bin correctly identifies variants unconfirmed with Sanger sequencing”. For this test, true positives were calculated as unconfirmed variants in the “low quality” (LQ) bin, false positives as confirmed variants in the LQ bin, true negatives as confirmed variants in the “high quality” (HQ) bin and false negatives as unconfirmed variants in the HQ bin.

Based on this, sensitivity (recall) was calculated as the number of unconfirmed variants in the LQ bin divided by the total number of unconfirmed variants, precision was calculated as the same number (unconfirmed variants in the LQ bin) divided by the size of the LQ bin (total number of the unconfirmed variants). F_1_-score was subsequently calculated as the harmonic mean of precision and recall and used as a metric that balances these values.

A reverse test (Test #2) was also formulated as “High quality bin correctly identifies variants confirmed by Sanger sequencing” which allows more intuitive definition of true and false positives but is worse suited for the task at hand. True and false positives for this test were calculated as confirmed and unconfirmed variants in the HQ bin respectively, false negatives and true negatives as confirmed and unconfirmed variants in the LQ bin, respectively. Statistical parameters based on these definitions were calculated accordingly and presented in Supplement.

## Electronic supplementary material

Below is the link to the electronic supplementary material.

Supplementary Material 1

## Data Availability

The data that support the findings of this study are available from Evogen LLC but restrictions apply to the availability of these data, which were used under license for the current study, and so are not publicly available. Data are however available from the authors upon reasonable request and with permission of Evogen LLC. Correspondence on the matter should be addressed to Olesya Sagaydak (sagaydak@evogenlab.ru). The code for the statistical analysis is available at https://github.com/bogdanovvp/sanger-validation/.
