# Supplementary material for: Sanger validation of WGS variants
Source: Sci Rep. 2025 Jan 29;15:3621. doi: 10.1038/s41598-025-87814-x (PMC11779820; doi:10.1038/s41598-025-87814-x)
Supplement: Supplementary file 1 — Supplementary Material 1 [file 41598_2025_87814_MOESM1_ESM.docx]

# Contents

Contents 1

**Table S1.** Variant information on the variants, unconfirmed by Sanger sequencing 2

**Table S2**. Classification statistics for the dataset in this work based on different thresholds 3

**Table S3**. Classification statistics for the dataset from Zheng, J. et al. (2019) based on different thresholds 4

**Table S4**. Classification statistics for different panels of the dataset from Zheng, J. et al. (2019) using caller-agnostic parameters DP ≥ 15, AF ≥ 0.25 from this work 5

**Figure S1**. Distribution of confirmed and unconfirmed variants depending on AF and DP parameters for the dataset from Zheng, J. et al. (2019) 6

**Figure S2**. Distribution of confirmed and unconfirmed variants depending on AF and DP parameters as well as sequencer and target capture for the dataset from Zheng, J. et al. (2019) – low DP range 7

**Figure S3**. Distribution of confirmed and unconfirmed variants depending on AF and DP parameters as well as sequencer and target capture for the dataset from Zheng, J. et al. (2019) – high DP range 8

# **Table S1.** Variant information on the variants, unconfirmed by Sanger sequencing

| CHROM | POS | ID | REF | ALT | QUAL | FILTER | INFO | FORMAT | SAMPLE |
| --- | --- | --- | --- | --- | --- | --- | --- | --- | --- |
| chr2 | 178658476 | rs1039780966 | C | T | 65.54 | PASS | AC=1;AF=0.500;AN=2;AS_BaseQRankSum=0.200;AS_FS=0.000;AS_MQ=27.93;AS_MQRankSum=1.600;AS_QD=13.20;AS_ReadPosRankSum=-1.100;AS_SOR=1.179;BaseQRankSum=0.253;DB;DP=5;ExcessHet=0.0000;FS=0.000;MLEAC=1;MLEAF=0.500;MQ=26.07;MQRankSum=1.65;QD=13.13;ReadPosRankSum=-1.036e+00;SOR=1.179 | GT:AD:DP:GQ:PL | 0/1:2,3:5:37:73,0,37 |
| chr6 | 156779110 | rs1778971493 | C | G | 38.64 | HardFiltered | AC=1;AF=0.500;AN=2;AS_BaseQRankSum=-0.400;AS_FS=3.233;AS_MQ=60.00;AS_MQRankSum=0.000;AS_QD=3.90;AS_ReadPosRankSum=1.900;AS_SOR=2.419;BaseQRankSum=-3.520e-01;DB;DP=11;ExcessHet=0.0000;FS=3.233;MLEAC=1;MLEAF=0.500;MQ=60.00;MQRankSum=0.00;QD=3.86;ReadPosRankSum=1.96;SOR=2.419 | GT:AD:DP:GQ:PL | 0/1:7,3:10:46:46,0,139 |
| chr12 | 51721774 | rs1942069105 | C | T | 30.64 | HardFiltered | AC=1;AF=0.500;AN=2;AS_BaseQRankSum=0.600;AS_FS=2.780;AS_MQ=60.00;AS_MQRankSum=0.000;AS_QD=1.41;AS_ReadPosRankSum=-0.700;AS_SOR=0.916;BaseQRankSum=0.672;DB;DP=25;ExcessHet=0.0000;FS=2.780;MLEAC=1;MLEAF=0.500;MQ=60.00;MQRankSum=0.00;QD=1.39;ReadPosRankSum=-6.220e-01;SOR=0.916 | GT:AD:DP:GQ:PL | 0/1:19,3:22:38:38,0,574 |
| chr19 | 41978752 | . | A | T | 31.64 | HardFiltered | AC=1;AF=0.500;AN=2;AS_BaseQRankSum=-1.000;AS_FS=0.000;AS_MQ=60.00;AS_MQRankSum=0.800;AS_QD=2.13;AS_ReadPosRankSum=-0.900;AS_SOR=1.179;BaseQRankSum=-5.050e-01;DP=18;ExcessHet=0.0000;FS=2.331;MLEAC=1;MLEAF=0.500;MQ=59.73;MQRankSum=0.949;QD=2.11;ReadPosRankSum=-6.080e-01;SOR=1.609 | GT:AD:DP:GQ:PL | 0/1:12,3:16:39:39,0,332 |
| chrX | 154364639 | . | G | C | 39.64 | PASS | AC=1;AF=0.500;AN=2;AS_BaseQRankSum=-0.700;AS_FS=0.000;AS_MQ=60.00;AS_MQRankSum=0.000;AS_QD=10.00;AS_ReadPosRankSum=-1.400;AS_SOR=0.105;BaseQRankSum=-6.740e-01;DP=4;ExcessHet=3.0103;FS=0.000;MLEAC=1;MLEAF=0.500;MQ=60.00;MQRankSum=0.00;QD=9.91;ReadPosRankSum=-1.383e+00;SOR=0.105 | GT:AD:DP:GQ:PL | 0/1:2,2:4:47:47,0,58 |

# **Table S2**. Classification statistics for the dataset in this work based on different thresholds

| Quality thresholds | “High quality” variants (unconfirmed variants) | “Low quality” variants (unconfirmed variants) | Test #1 “LQ bin identifies unconfirmed variants” | | | Test #2 “HQ bin identifies confirmed variants” | | |
| --- | --- | --- | --- | --- | --- | --- | --- | --- |
|  |  |  | Sensitivity (Recall) | Precision | F_1_-score | Sensitivity (Recall) | Precision | F_1_-score |
| DP ≥ 35,  AF ≥ 0.35[^2^](https://paperpile.com/c/J9fdvD/kLg7W) | 738 (0) | 1018 (5) | 100 % | 0.5 % | 0.010 | 42.1 % | 100 % | 0.593 |
| DP ≥ 30  QUAL ≥ 30[^13^](https://paperpile.com/c/J9fdvD/1cC6) | 1088 (0) | 668 (5) | 100 % | 0.7 % | 0.015 | 62.1 % | 100 % | 0.766 |
| DP ≥ 20[^12^](https://paperpile.com/c/J9fdvD/CeIa) | 1549 (1) | 207 (4) | 80.0 % | 1.9 % | 0.038 | 88.4 % | 99.9 % | 0.938 |
| DP ≥ 20  AF ≥ 0.3 FILTER = PASS  QUAL > 300[^5^](https://paperpile.com/c/J9fdvD/Do9tv) | 1357 (0) | 399 (5) | 100 % | 1.3 % | 0.025 | 77.5 % | 100 % | 0.873 |
| DP ≥ 20 AF ≥ 0.2 FILTER = PASS  QUAL ≥ 100[^8^](https://paperpile.com/c/J9fdvD/SBAP0) | 1546 (0) | 210 (5) | 100 % | 2.4 % | 0.047 | 88.3 % | 100 % | 0.938 |
| DP ≥ 20 AF ≥ 0.2 | 1547 (0) | 209 (5) | 100 % | 2.4 % | 0.047 | 88.3 % | 100 % | 0.938 |
| **DP ≥ 15 AF ≥ 0.25** | **1672 (0)** | **84 (5)** | **100 %** | **6.0 %** | **0.112** | **95.5 %** | **100 %** | **0.977** |
| **QUAL ≥ 100** | **1735 (0)** | **21 (5)** | **100 %** | **23.8 %** | **0.385** | **99.1 %** | **100 %** | **0.995** |

**Bold** font denotes thresholds suggested in this work

# **Table S3**. Classification statistics for the dataset from Zheng, J. et al. (2019) based on different thresholds

| Quality thresholds | “High quality” variants (unconfirmed variants) | “Low quality” variants (unconfirmed variants) | Test #1 “LQ bin identifies unconfirmed variants” | | | Test #2 “HQ bin identifies confirmed variants” | | |
| --- | --- | --- | --- | --- | --- | --- | --- | --- |
|  |  |  | Sensitivity (Recall) | Precision | F_1_-score | Sensitivity (Recall) | Precision | F_1_-score |
| ***DP ≥ 35***  ***AF ≥ 0.35***[***^2^***](https://paperpile.com/c/J9fdvD/kLg7W) | ***6939 (0)*** | ***662 (236)*** | ***100 %*** | ***35.6 %*** | ***0.526*** | ***94.2 %*** | ***100 %*** | ***0.970*** |
| DP ≥ 35  AF ≥ 0.34 | 6976 (4) | 625 (232) | 98.3 % | 37.1 % | 0.539 | 94.7 % | 99.9 % | 0.972 |
| DP ≥ 20[^12^](https://paperpile.com/c/J9fdvD/CeIa) | 7450 (176) | 151 (60) | 25.4 % | 39.7 % | 0.310 | 98.8 % | 97.6 % | 0.982 |
| DP ≥ 20 AF ≥ 0.2 | 7297 (47) | 304 (189) | 80.1 % | 62.2 % | 0.700 | 98.4 % | 99.4 % | 0.989 |
| **DP ≥ 15 AF ≥ 0.25** | **7295 (30)** | **306 (206)** | **87.3 %** | **67.3 %** | **0.760** | **98.6 %** | **99.6 %** | **0.991** |

**Bold** font denotes thresholds suggested in this work. ***Bold italic*** font denotes thresholds from the original paper.

# **Table S4**. Classification statistics for different panels of the dataset from Zheng, J. et al. (2019) using caller-agnostic parameters DP ≥ 15, AF ≥ 0.25 from this work

| Panel name (Number of exons) | “High quality” variants (unconfirmed variants) | “Low quality” variants (unconfirmed variants) | Test #1 “LQ bin identifies unconfirmed variants” | | | Test #2 “HQ bin identifies confirmed variants” | | |
| --- | --- | --- | --- | --- | --- | --- | --- | --- |
|  |  |  | Sensitivity (Recall) | Precision | F_1_-score | Sensitivity (Recall) | Precision | F_1_-score |
| HC (1734) | 332 (0) | 5 (0) | - | - | - | - | - | - |
| HD (4899) | 1567 (2) | 65 (59) | 96.7 % | 90.7 % | 0.937 | 99.6 % | 99.9 % | 0.997 |
| 4.8M (17108) | 2002 (1) | 64 (34) | 97.1 % | 53.1 % | 0.687 | 98.5 % | 99.95 % | 0.992 |
| C2181 (31632) | 2607 (11) | 104 (65) | 85.5 % | 62.5 % | 0.722 | 98.5 % | 99.6 % | 0.990 |
| BGI_Exo (197748) | 787 (16) | 68 (48) | 75.0 % | 70.6 % | 0.727 | 97.5 % | 98.0 % | 0.977 |

# **Figure S1**. Distribution of confirmed and unconfirmed variants depending on AF and DP parameters for the dataset from Zheng, J. et al. (2019)


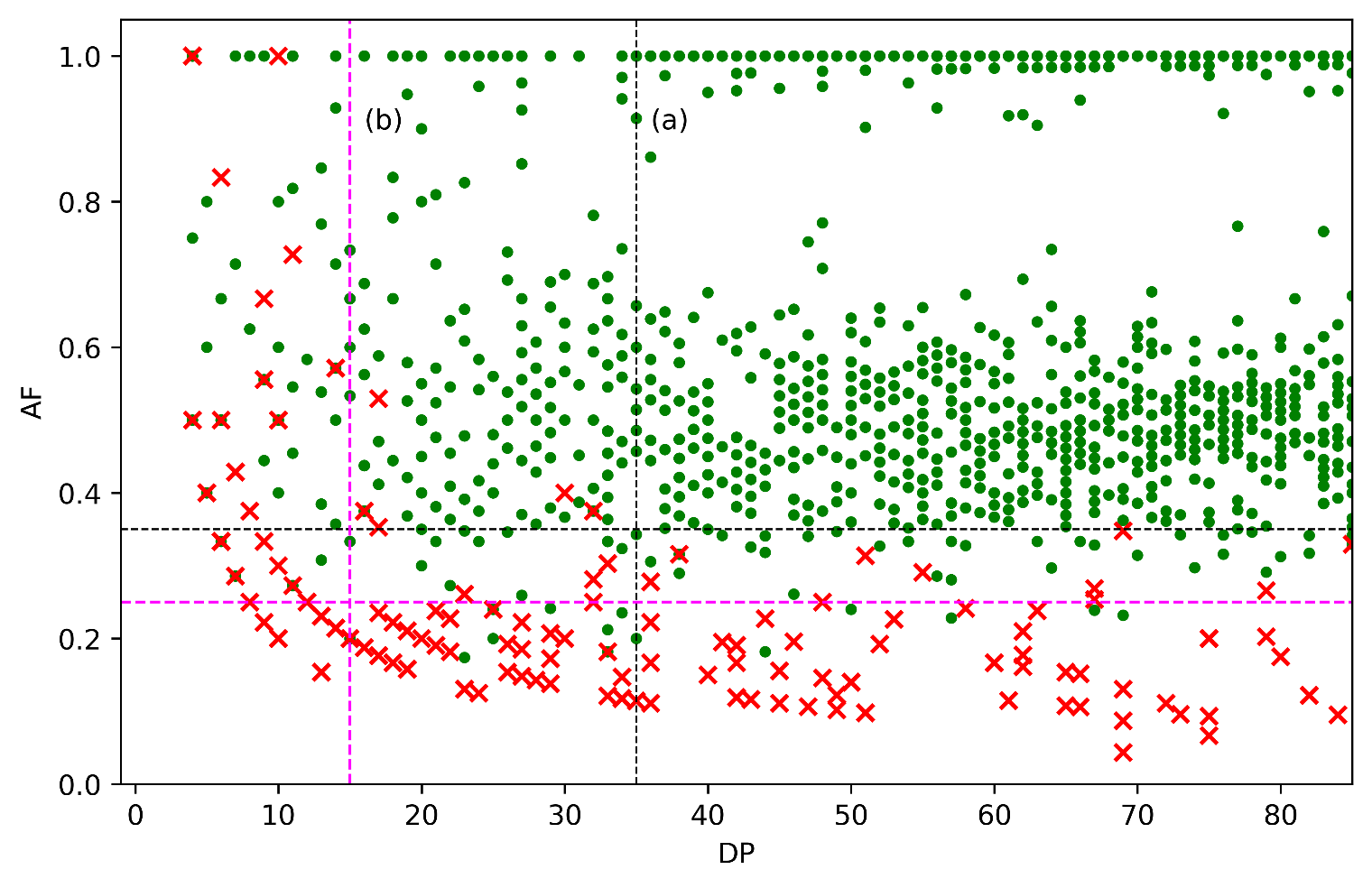


1. Thresholds from the original work (b) Caller-agnostic thresholds suggested in this work

# **Figure S2**. Distribution of confirmed and unconfirmed variants depending on AF and DP parameters as well as sequencer and target capture for the dataset from Zheng, J. et al. (2019) – low DP range

| 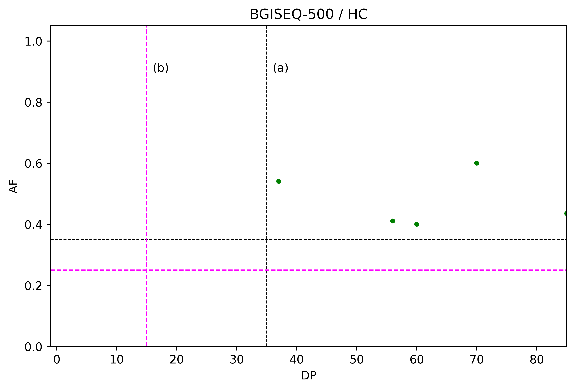 | 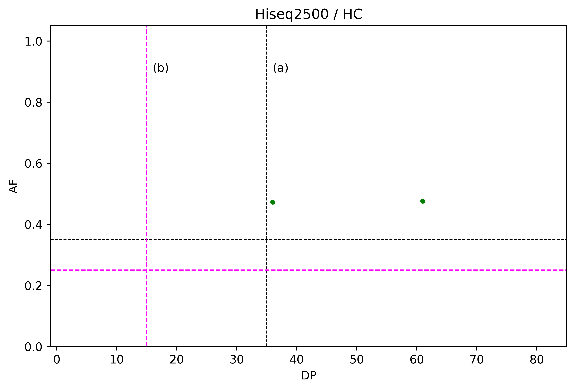 |
| --- | --- |
| 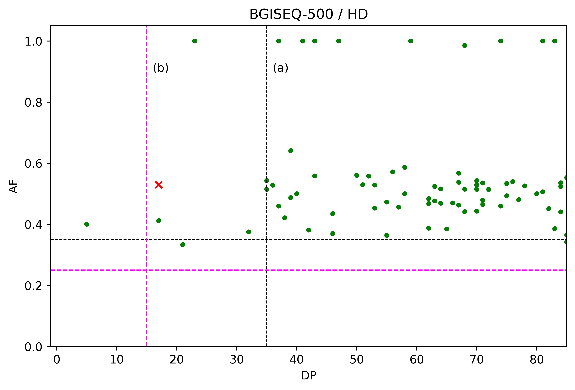 | 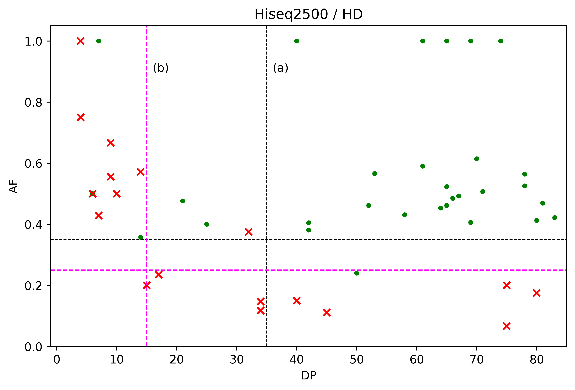 |
| 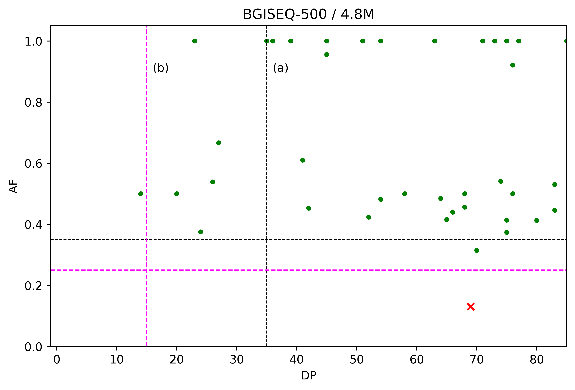 | 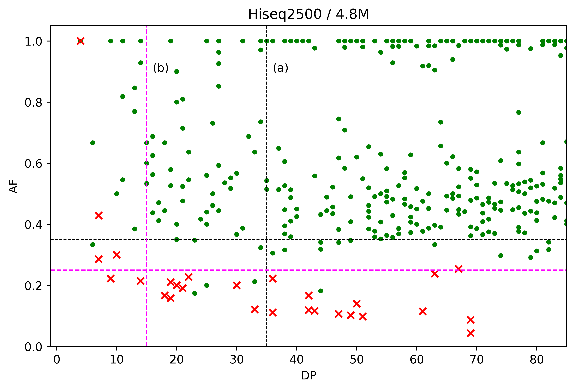 |
| 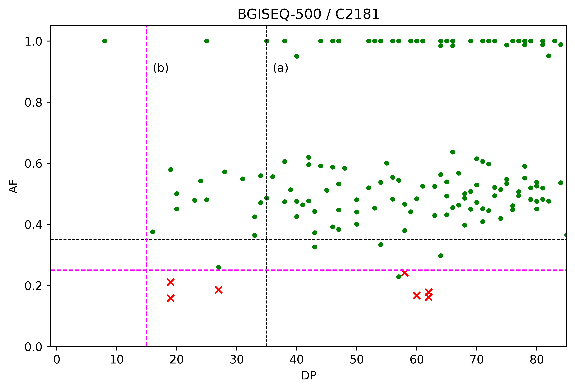 | 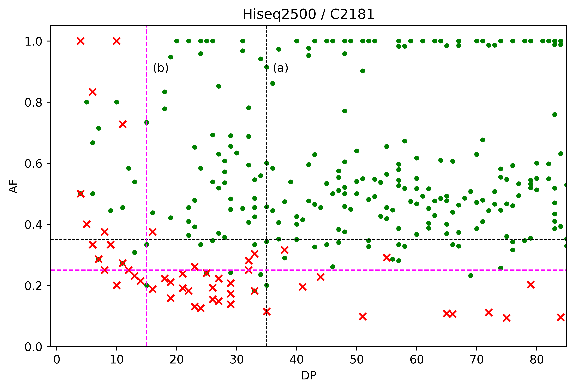 |
| 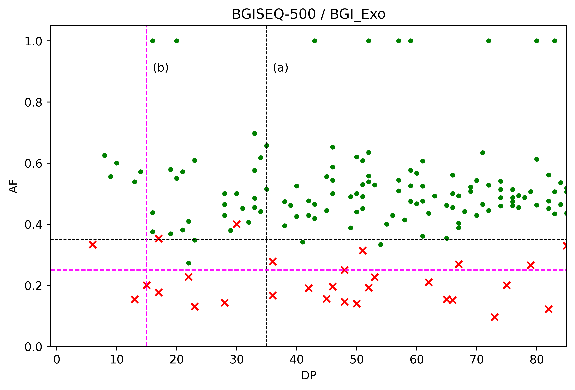 | 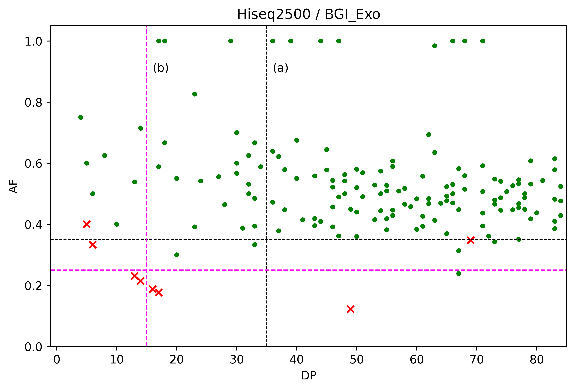 |

# **Figure S3**. Distribution of confirmed and unconfirmed variants depending on AF and DP parameters as well as sequencer and target capture for the dataset from Zheng, J. et al. (2019) – high DP range

| 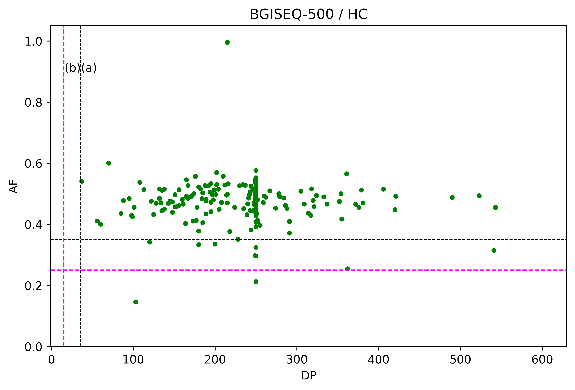 | 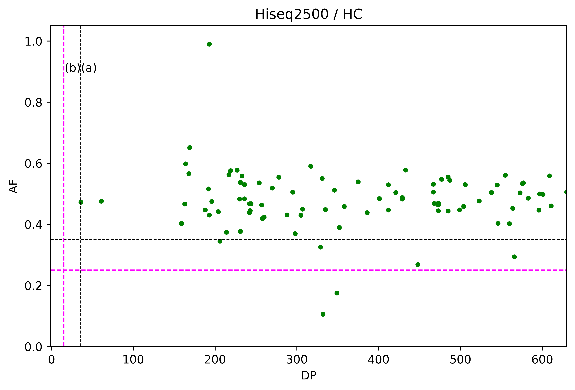 |
| --- | --- |
| 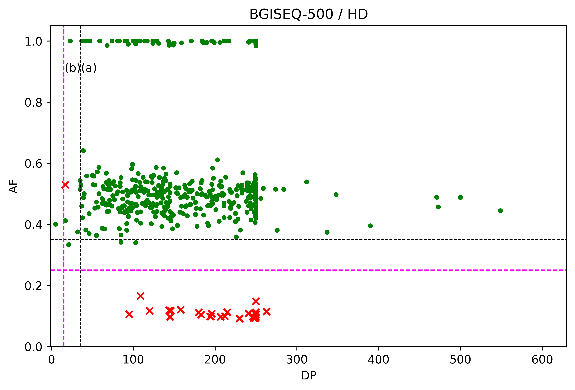 | 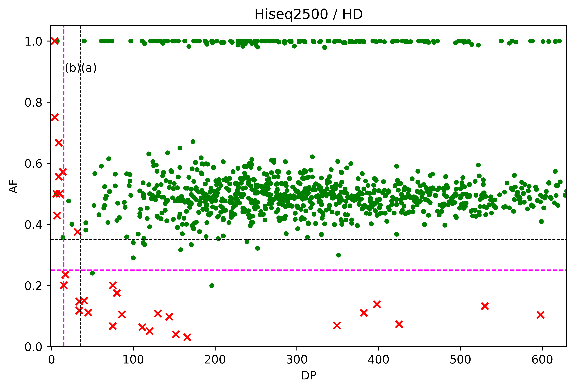 |
| 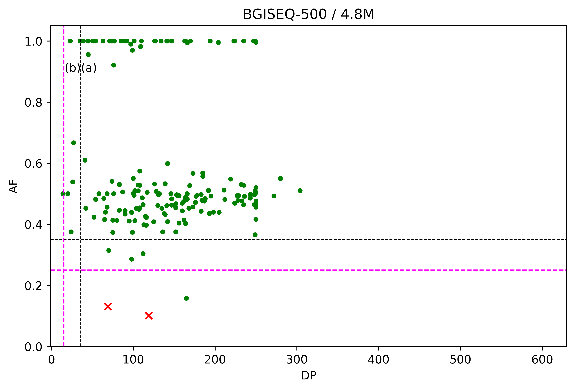 | 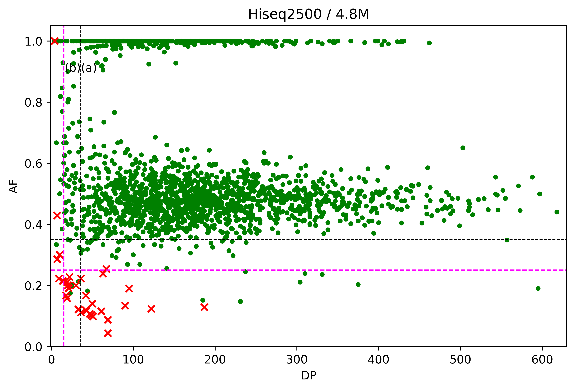 |
| 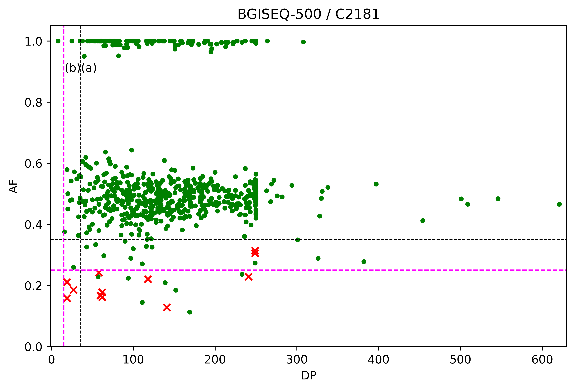 | 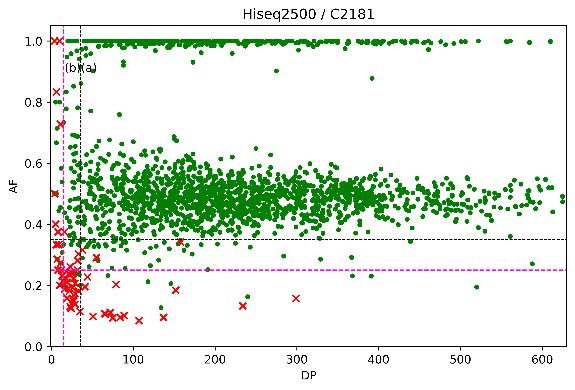 |
| 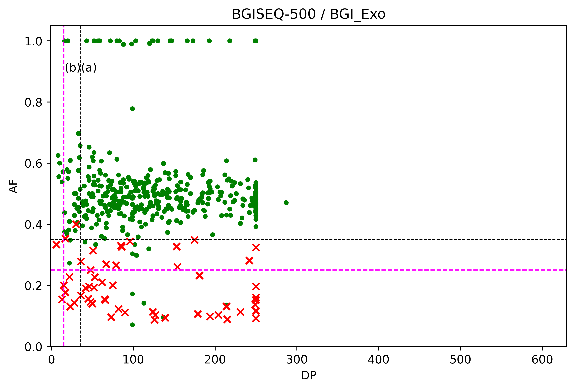 | 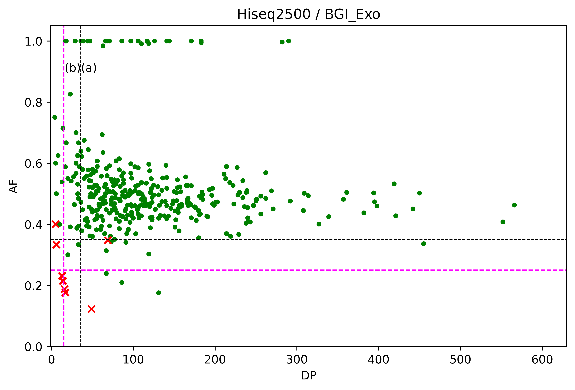 |
